# Impact of Age and Biological Sex on Cerebrovascular Reactivity in Adult Moderate/Severe Traumatic Brain Injury: An Exploratory Analysis

**DOI:** 10.1089/neur.2021.0039

**Published:** 2021-11-09

**Authors:** Carleen Batson, Logan Froese, Alwyn Gomez, Amanjyot Singh Sainbhi, Kevin Y. Stein, Arsalan Alizadeh, Frederick A. Zeiler

**Affiliations:** ^1^ Department of Human Anatomy and Cell Science, Department of Surgery, Rady Faculty of Health Sciences, University of Manitoba, Winnipeg, Manitoba, Canada.; ^2^ Biomedical Engineering, Faculty of Engineering, University of Manitoba, Winnipeg, Manitoba, Canada.; ^3^ Section of Neurosurgery, Department of Surgery, Rady Faculty of Health Sciences, University of Manitoba, Winnipeg, Manitoba, Canada.; ^4^ Centre on Aging, University of Manitoba, Winnipeg, Manitoba, Canada.; ^5^ Division of Anaesthesia, Department of Medicine, Addenbrooke's Hospital, University of Cambridge, Cambridge, United Kingdom.

**Keywords:** aging, biological sex, cerebrovascular reactivity, signal processing, TBI

## Abstract

Age and biological sex are two potential important modifiers of cerebrovascular reactivity post-traumatic brain injury (TBI) requiring close evaluation for potential subgroup responses. The goal of this study was to provide a preliminary exploratory analysis of the impact of age and biological sex on measures of cerebrovascular function in moderate/severe TBI. Forty-nine patients from the prospectively maintained TBI database at the University of Manitoba with archived high-frequency digital cerebral physiology were evaluated. Cerebrovascular reactivity indices were derived as follows: PRx (correlation between intracranial pressure [ICP] and mean arterial pressure [MAP]), PAx (correlation between pulse amplitude of ICP [AMP] and MAP), and RAC (correlation between AMP and cerebral perfusion pressure [CPP]). Time above clinically significant thresholds for each index was calculated over different periods of the acute intensive care unit stay. The association between PRx, PAx, and RAC measures with age was assessed using linear regression, and an age trichotomization scheme (<40, 40–60, >60) using Kruskal-Wallis testing. Similarly, association with biological sex was tested using Mann-Whitney *U* testing. Biological sex did not demonstrate an impact on any measures of cerebrovascular reactivity. Linear regression between age and PAx and RAC demonstrated a statistically significant positive linear relationship. Median PAx and RAC measures between trichotomized age categories demonstrated statistically significant increases with advancing age. The PRx failed to demonstrate any statistically significant relationship with age in this cohort, suggesting that in elderly patients with controlled ICP, PAx and RAC may be better metrics for detecting impaired cerebrovascular reactivity. Biological sex appears to not be associated with differences in cerebrovascular reactivity in this cohort. The PRx performed the worst in detecting impaired cerebrovascular reactivity in those with advanced age, where PAx and RAC appear to have excelled. Future work is required to validate these findings and explore the utility of different cerebrovascular reactivity indices.

## Introduction

Traumatic brain injury (TBI) is one of the leading causes of morbidity and death in young people globally and occurs across a spectrum of severity. The yearly incidence of TBI is 69 million worldwide, leading to significant disability, mortality rates (∼30–40% in patients with severe TBI), and cost (US $400 billion).^1,2^ In TBI, the primary injury refers to the inciting event of trauma (i.e., external force), which can potentially lead to secondary injuries referred to as the cascade of molecular and cellular events leading to ongoing neural injury and death.^3,4^

More specific, the secondary injury involves a sequelae of events at the cellular, physiological, metabolic, and molecular levels, including disrupted cerebral autoregulation, impaired extracellular homeostasis, activation of inflammatory mediators, change to anaerobic metabolism, mitochondrial failure, and such.^3,5–7^ Continued efforts are being made in the field to try to optimize prompt management to allay the severity of secondary brain injury, especially in patients with moderate to severe TBI, because this causes substantial loss of neurological tissue, leading to poor outcomes.^3,5–7^

Cerebral autoregulation refers to the change in cerebral blood vessel tone in response to changes in cerebral perfusion pressure (CPP) to maintain constant cerebral blood flow.^8^ Although direct continuous bedside assessment of cerebral autoregulation is complex, recent advances in biomedical signal processing of data from multi-modal monitoring (MMM) in moderate/severe TBI have facilitated the development of surrogate measures, some of which are referred to as cerebrovascular reactivity metrics. It must be acknowledged, however, that these derived metrics encompass additional cerebral physiological information beyond that of pure cerebrovascular reactivity.

The pressure reactivity index (PRx) is the exemplar and is a continuous derived metric of cerebrovascular reactivity based on the moving Pearson correlation coefficient between slow waves of intracranial pressure (ICP) and mean arterial pressure (MAP).^9^ Impaired cerebrovascular reactivity, as measured through PRx, in moderate/severe TBI has emerged as one of the most prevalent forms of impaired cerebral physiology during the acute phase after injury, leading to ongoing secondary insults.^10,11^

Further, current guideline-based therapeutic strategies have failed to impact the incidence and duration of impaired cerebrovascular reactivity after moderate/severe TBI.^12–16^ As such, more work needs to be done to truly understand the drivers of impaired cerebrovascular reactivity and potential pathways for therapeutic intervention.^17^

At the core of our current knowledge gap regarding cerebrovascular reactivity in moderate/severe TBI and its drivers are the impact of age and biological sex. We know both age and biological sex are critical factors in the overall outcome of patients with TBI. Age alone is one of the major drivers of prognosis and is a crucial component of existing standard prognostic models in moderate/severe TBI. Preliminary data on the impact of age on cerebrovascular reactivity are limited, although it does suggest worse cerebrovascular reactivity post-TBI in older patients.^18,19^ As we age, the functioning of our arteries deteriorates, and the literature has shown that older persons tend to have the worst outcomes.^18,19^ Some reasons for the worsening in cerebrovascular response are chronic inflammation, accumulation of amyloid-based substances, and suboptimal neuronal repair mechanisms.^20,21^

At the same time, biological sex is known to modulate secondary injury responses, particularly concerning the presence of female sex hormones and their downstream effects on neural injury mitigation and repair. This is despite males dominating the TBI cohorts in terms of overall incidence. Females in their young years benefit from estrogen's protective vasodilatory and anti-inflammatory effects on the neurovasculature but lose this once menopausal.^22–24^ Finally, an interplay between age and sex can be possible as seen in older females with less estrogen and more deranged cerebrovascular reactivity.^23^

Our understanding of the role that age and sex play on continuous MMM cerebral physiology, including cerebrovascular reactivity metrics, however, is still in its infancy with very few focused studies to date.^18,19^ Further, previous work has focused on grand-averaged data and not insult burden metrics. Insult burden metrics are emerging as superior assessments for cerebral physiological dysfunction, where the amount of time spent above known physiological thresholds (example: Brain Trauma Foundation-based [BTF-based] and literature-defined critical thresholds for ICP of 20 mm Hg) display stronger associations with global outcomes, compared with grand averaged values over large epochs of recorded physiology.^25–27^ As such, a better understanding of insult burden is crucial if we are to transition toward personalized precision medicine in moderate/severe TBI care.

Our group performed a review, which is currently under consideration, of the existing literature to garner information on the age and biological sex-related impacts on continuously assessed cerebrovascular reactivity post-moderate/severe TBI, but few large studies were found. Such existing works examined overall grand averages of the data collected over extended periods, and lacked a more detailed analysis of age subgroups. Given the limited available literature, we aimed to explore the association between various age groups and biological sex and how these separately impact continuously measured cerebrovascular reactivity in adult patients with moderate/severe TBI during the acute phase of their intensive care unit (ICU) stay.

The working hypothesis is that cerebral autoregulation is an age-related process that begins in its immaturity during youth, matures in function, and then deteriorates with age. As such, it should naturally follow that such age-related variations would be seen in patients with moderate/severe TBI, where continuous cerebrovascular reactivity measures can be derived based on available invasive intracranial monitoring. Further, we hypothesized that younger females would have a slight degree of improved cerebrovascular reactivity versus younger males that would disappear after menopause in moderate/severe TBI.

## Methods

### Study type

This is a local retrospective evaluation of a prospectively maintained approved database of patients with moderate to severe TBI. The database contains demographic, injury, treatment, outcome information and high-frequency physiological signals from various MMM devices. Local research ethics board approval at the University of Manitoba is in place for all aspects of this database (H2017:181 and H2017:188). Similarly, we obtained ethics approval for retrospective access to the database for this project (H2020:118).

### Patient population

Patient data were entered into the database from January 2019 to December 2020. Inclusion criteria for this study were patients 16 years and older who had moderate to severe TBI with invasive ICP and arterial blood pressure (ABP) monitoring and who were admitted to the Health Sciences Centre Winnipeg–Surgical Intensive Care Unit (SICU). Patients who were younger than 16 years, those with mild TBI and those without invasive ICP monitoring were excluded from this study.

### Patient data collected

For the purpose of this study, we extracted general admission demographics for the patient cohort from the prospectively maintained database. Such demographic variables included: age, sex, admission pupillary response, admission Glasgow Coma Scale (GCS) total and motor subscore, and admission Marshall and Rotterdam computed tomography (CT) grade.

### Physiologic data acquisition

As part of the standard care locally for critically ill moderate/severe TBI, patients have ABP and ICP monitoring placed in keeping with standard BTF guidelines. The ABP was monitored via radial arterial lines while ICP was monitored via intraparenchymal strain gauge monitors (Codman ICP Microsensor; Codman & Shurtleff Inc., Raynham, MA). All physiology data were recorded in digital high-frequency time series (i.e., 100 Hz) using analogue-to-digital signal converters (Data Translations, DT9804 or DT9826), where applicable. These digital data were linked and stored in time series using Intensive Care Monitoring (ICM+) software (Cambridge Enterprise Ltd, Cambridge, UK).^28^

### Physiologic signal processing

Archived high-frequency physiological data from the patient cohort underwent the following steps for processing. The first step in preparing the data for analysis was artifact clearing whereby ICM+ software was used to remove these flaws manually. Such artifact clearing was conducted by a qualified specialist clinician experienced with all of the multi-modal cerebral physiological signals both in bedside care and the laboratory environment. All signal files were cleaned without knowledge of patient age or sex, given they were completely de-identified when stored in the signals database.

The CPP was calculated as MAP minus ICP. Next, pulse amplitude of ICP (AMP) was derived using Fourier analysis of the ICP pulse waveform. Subsequently, the full waveform data for all physiology were decimated using a non-overlapping moving average filter of 10-sec duration that facilitates focusing on the slow-wave frequency range associated with cerebral autoregulation. This 10-sec by 10-sec data were then used to derive continuously updated cerebrovascular reactivity and compensatory reserve measures. As an exemplar, PRx is calculated as the moving Pearson correlation coefficient between 30 consecutive 10-sec mean paired values of ICP and MAP. A similar process is used to calculate the other indices.

The following cerebrovascular reactivity indices were derived: PRx—correlation between slow waves of MAP and ICP,^29^ pulse amplitude index (PAx)—correlation between slow waves of AMP and MAP,^29^ and RAC (correlation [R] between slow-waves of AMP [A] and CPP [C]). Finally, the continuously updating compensatory reserve index, RAP (correlation between slow-waves of AMP and ICP),^30^ was also derived to facilitate surrogate evaluation of brain compliance. All recorded and derived signals were output in minute averages into comma-separated values (CSV) datasets for further analysis. Such minute by minute data update frequency for further analytics is considered standard within the field of signal analytics, using high-frequency cerebral physiological signals in moderate/severe TBI.^31^

### Physiologic data post-processing

The data were sorted into two main datasheets—i.e., the first (1st) 72 h of recording and the entire recording period. The 1st 72 h are significant being the window where most acute physiology changes would occur and where aggressive treatment occurs post-TBI.^26,32,33^ Also, this is the period where autoregulation is impaired and blood flow is affected by various external and internal mechanisms leaving the cerebrovasculature ischemia prone.^34^ As such, we wished to determine whether focusing on only the “acute” phase of cerebral physiological dysfunction would lead to different results, compared with the entire recording period available.

Such analytic strategies, utilizing the 1st 72 h of data, have been employed in various other studies pertaining to cerebral physiological dysfunction in moderate/severe TBI.^33,35–38^ We wish to follow similar analytic strategies. The two data sheets serve to evaluate the differences between the acute physiology versus the entire recording period. This was done to avoid the pitfalls of previous literature regarding grand averages of cerebrovascular reactivity metrics, and we derived insult burden metrics for key physiological measures. This was done across all of the data sheets described above. Such insult burden metrics were defined as the percent time spent above literature-defined thresholds for ICP, CPP, PRx, PAx, and RAC. These derived variables included:

1. % time with ICP above 20 and 22 mm Hg. These are BTF-based ICP treatment thresholds.^9,25^
2. % time with CPP below 60 mm Hg and above 70 mm Hg. These are BTF-based CPP thresholds.^9,25^
3. % time with PRx above 0, +0.25, and +0.35. These are literature-defined critical thresholds associated with outcomes in TBI.^9,25^
4. % time with PAx above 0 and +0.25. These are literature-defined critical thresholds associated with outcomes in TBI.^35^
5. % time with RAC above -0.10 and -0.05. These are literature-defined critical thresholds associated with outcomes in TBI.^35^

### Statistical analysis

Statistical analysis was performed using the R statistical software (R Core Team [2020]). R: A language and environment for statistical computing. R Foundation for Statistical Computing, Vienna, Austria).^39^ Patient demographics and physiological variables for both data sheets were summarized using descriptive statistics; mean/standard deviation (SD) and median/interquartile range (IQR) averages, where appropriate. Box plots were used to aid with the visualization of the data. Shapiro-Wilks test was performed on the continuous variables from these two data sets to test for normality, with all physiological variables demonstrating non-parametric nature. Alpha for all statistical testing described was set at 0.001 for significance after correction for multiple comparisons was done using the Bonferroni method. The impact of biological sex on the continuously measured metrics (namely ICP, MAP, CPP, PRx, PAx, RAC, and RAP) was assessed by comparing values between males and females, using the Mann-Whitney *U* test.

Age was assessed through three methods: linear regression, dichotomization, and trichotomization categorical analysis. First, using age as a continuous variable, it was compared with the physiology variables using both Pearson and Kendall Tau correlation, with linear regression modeling. Data visualization was aided using scatterplots, linear regression plotting (95% confidence interval [CI]). Next, the patient cohort was split based on age dichotomization (i.e., age <60 vs. age ≥60 years) and trichotomization (i.e., age <40, age 40–60, and age >60 years). The cutoff for the age of 60 is a common cutoff for outcomes in various neuropathological and critical care states, with those above the age of 60 demonstrating poorer long-term outcomes.^25,40–42^

The trichotomization scheme for age was to evaluate the hypothesis of age-related maturation of cerebrovascular function in this cohort, with the rationale for the age of 60 as a cutoff provided above. Regarding the age of 40, this was used as a cutoff because it was noted in the literature that there is potentially more unfavorable outcomes across the age groups centered on 40 years with persons <40 years likely to experience good recovery as opposed to persons >40 years.^43–45^ Comparison of physiologic metrics between these groups was conducted using Mann-Whitney *U* and Kruskal-Wallis testing, respectively. Age dichotomization failed to demonstrate any statistically significant relationships with cerebral physiological variables. As such, categorical age analysis reporting in the results will focus mainly on the trichotomization categorical analysis.

## Results

### Patient demographics

Patient data were entered into the database from January 2019 to December 2020. From here, 49 patients were identified as eligible for the study—42 males and seven females with a median age of 42 (IQR: 24.0–54.0). All patients sustained moderate (GCS score 9–12) to severe TBI (GCS score <9) necessitating ICU admission, with a median GCS score of 7 (IQR: 5.0–9.0). General patient demographics, summarized in means/medians, can be found in Table 1. Similarly, recorded cerebral physiological variables, summarized in medians and IQRs, can be found in Table 2 for both the 1st 72 h of physiology (the acute phase) and the entire recording period. The median duration of recorded physiology was 2.67 days (IQR: 1.49–4.96). All physiology variables were non-parametric.

**Table 1.** Patients Demographic Information

Demographic Information				
	Mean	SD	Median	IQR
Age (years)	41.6	17.0	42.0	24.0–54.0
Best admission GCS	7.2	3.0	7.0	5.0–9.0
Best admission GCS – Motor	4.0	1.7	5.0	3.0–5.0

	Unilateral reactive	Unilateral Unreactive	Bilateral reactive	Bilateral Unreactive
Pupils	0	10	31	8
	*Males*		*Females*	
Sex	42		7	
Admission Marshall CT grade:	Males		Females	
II	2		-	
III	11		3	
IV	9		1	
V	20		3	
ROTTERDAM CT grade:	Males		Females	
I	1		-	
II	3		-	
III	5		1	
IV	12		2	
V	11		2	
VI	10		2	
	*Yes*		*No*	
Pre-hospital Hypoxia	*23*		*26*	
Admission CT – tSAH	47		2	
Admission CT – EDH	5		44	

CT, computed tomography; EDH, epidural hematoma; GCS, Glasgow Coma Scale; IQR, interquartile range, SD, standard deviation, tSAH, traumatic subarachnoid hemorrhage.

**Table 2.** Cerebral Physiology for the First 72 hours and Entire Recording Period

Physiological variables	1st 72 h of data		Entire recording period	
	Median	IQR	Median	IQR
ICP (mm Hg)	10.7	7.3–13.3	10.9	7.2–13.6
% Time ICP >20 mm Hg	2.3	0.9–7.3	2.8	0.9–10.5
% Time ICP >22 mm Hg	1.7	0.33.8	1.7	0.3–5.4
MAP (mm Hg)	81.1	77.5–88.3	81.7	77.5–86.8
CPP (mm Hg)	71.6	64.4 − 76.4	70.9	64.4–74.6
% Time CPP >70 mm Hg	57.4	40.8–73.6	56.4	42.2–73.4
% Time CPP <60 mm Hg	8.6	2.9–19.1	10.8	3.2–21.2
PRx (a.u.)	0.143	0.057–0.343	0.147	0.030–0.329
% Time PRx >0	67.0	51.8–82.9	68.4	51.0–83.2
% Time PRx >0.25	41.1	28.7–64.3	41.2	24.9–61.8
% Time PRx >0.35	31.2	18.6–52.4	31.2	17.4–50.4
PAx (a.u.)	0.001	-0.077–0.243	0.018	-0.073–0.221
% Time PAx >0	50.6	38.1–76.7	51.6	37.6–73.8
% Time PAx >0.25	24.1	16.2–53.0	24.8	16.2–51.0
RAC (a.u.)	-0.161	-0.309–0.020	-0.191	(-0.317)–(-0.019)
% Time RAC > -0.10	44.3	27.5–67.4	40.5	26.7–63.3
% Time RAC > -0.05	38.6	24.2–63.3	36.3	23.0–57.7
RAP (a.u.)	0.595	0.411–0.740	0.595	0.411–0.746
% Time RAP >0.4	76.5	57.6–86.3	76.5	57.4–86.3

a.u., arbitrary units; AMP, pulse amplitude of ICP; CPP, cerebral perfusion pressure; ICP, intracranial pressure; IQR, interquartile range; MAP, mean arterial pressure; mm Hg, millimeters of mercury; PAx, pulse amplitude index; PRx, pressure reactivity index; RAC, correlation (R) between slow-waves of AMP (A) and CPP (C); RAP, compensatory reserve index.

### Daily insult burden of impaired cerebrovascular reactivity

**FIG. 1.**
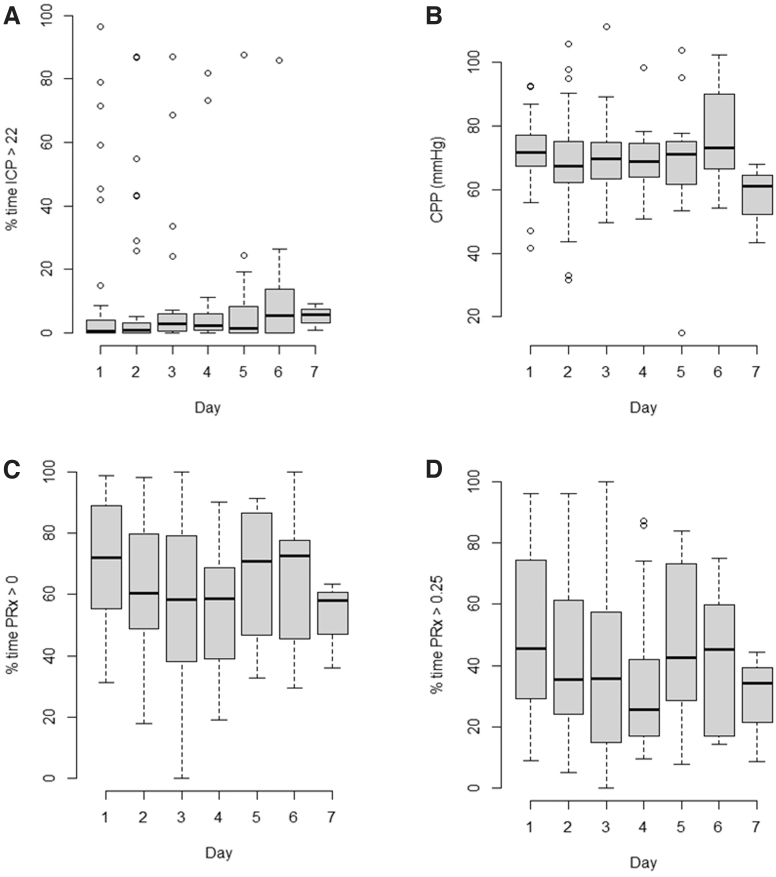
Box plots of daily recordings of the first seven days: % time intracranial pressure (ICP) >22 mm Hg, mean cerebral perfusion pressure (CPP), % time pressure reactivity index (PRx) >0, and % time PRx > +0.25. Using a Kruskal-Wallis test to compare the different days, we found no statistically significant difference between days for any of the physiology displayed. The number of patient data for each day: day 1 has 49, day 2 has 42, day 3 has 32, day 4 has 21, day 5 has 18, day 6 has 9, and day 7 has 4. Panel A, % time with ICP >22 mm Hg; Panel B, mean CPP; Panel C, % time with PRx >0; Panel D, % time with PRx > +0.25.

To aid with understanding the degree of impaired cerebrovascular reactivity seen in our cohort, we analyzed the % time of index impairment over the first seven days of their ICU stay. Overall, during the first seven days, ICP and CPP were well controlled, with limited time spent with ICP above 22 mm Hg and CPP well maintained within the guideline suggested range of 60–70 mm Hg. Most patients, however, spent the first week of their ICU stay with a daily PRx value above 0 and +0.25 for the majority of each day. Kruskal-Wallis testing, assessing for differences between days for cerebral physiology, failed to demonstrate a significant difference (*p* > 0.05 for all), confirming that the median daily values remained relatively constant. Figure 1 displays box plots for the first seven days, highlighting % time with ICP >22 mm Hg (Fig. 1A), mean CPP (Fig. 1B), % time with PRx >0 (Fig. 1C), and % time with PRx > +0.25 (Fig. 1D).

### Sex analysis

To assess the difference in physiological responses based on biological sex, we compared male and female patients. Data presented in Table 3 display medians/IQRs and the Mann-Whitney *U* test *p* values for the comparison of the median physiological variables between males and females for the 1st 72 h of data. Supplementary Appendix SA1 shows a similar table for the entire recording period, with similar findings displayed.

**Table 3.** Cerebral Physiology Based on Biological Sex: Medians/Interquartile Ranges and Mann-Whitney *U* Testing of the First 72 Hours of Recording

Physiological Variable	Males (n = 42)		Females (*n* = 7)		Mann-Whitney* U *Test *p*
	Median	IQR	Median	IQR	
ICP (mm Hg)	9.1	6.9–12.7	17.7	12.3–25.3	**0.007**
% Time ICP >20 mm Hg	2.3	0.4–6.1	38.5	6.4–84.3	**0.012**
% Time ICP >22 mm Hg	1.5	0.2–2.7	31.8	3.6–78.2	**0.010**
MAP (mm Hg)	81.0	77.5 − 89.4	81.9	77.5–85.4	0.834
CPP (mm Hg)	71.7	67.5–77.0	62.3	57.5–66.0	**0.016**
% Time CPP >70 mm Hg	57.9	44.5–74.5	29.9	20.6–39.6	**0.007**
% Time CPP <60 mm Hg	7.2	2.6–17.0	18.6	12.9–50.9	0.055
PRx (a.u.)	0.143	0.058–0.278	0.073	-0.014–0.664	1.000
% Time PRx >0	68.3	52.4–82.8	58.2	45.4–86.0	0.710
% Time PRx >0.25	41.4	29.1–57.3	35.9	23.3–82.8	0.977
% Time PRx >0.35	31.9	18.8–45.1	25.9	16.6–80.2	0.932
PAx (a.u.)	0.005	-0.065–0.220	-0.008	-0.078–0.495	0.753
% Time PAx >0	50.7	38.1–75.7	47.9	41.4–82.9	0.932
% Time PAx >0.25	24.4	16.–51.0	23.8	17.9–75.5	0.627
RAC (a.u.)	-0.160	(-0.287)–(-0.017)	-0.192	-0.420–0.309	0.932
% Time RAC > -0.10	45.3	29.5–62.0	38.5	21.2–78.5	0.886
% Time RAC > -0.05	38.8	26.5– 5.8	33.9	19.4–76.1	0.954
RAP (a.u.)	0.583	0.406–0.717	0.658	0.615–0.801	0.084
% Time RAP >0.4	75.1	57.5–83.3	85.1	79.2–94.1	0.069
Age (years)	44.5	27.5–54.8	24.0	23.0–34.0	0.092
Best admission GCS	7.0	5.3–9.0	7.0	3.0–7.5	0.267
Best admission GCS – Motor	5.0	3.0–5.0	5.0	1.0–5.0	0.572
Rotterdam CT grade	4.5	4.0–5.0	5.0	4.0–5.5	0.638

a.u., arbitrary units; AMP, pulse amplitude of ICP; CPP, cerebral perfusion pressure; CT, computed tomography; GCS, Glasgow Coma Scale; ICP, intracranial pressure; IQR, interquartile range; MAP, mean arterial pressure; mm Hg, millimeters of mercury; PAx, pulse amplitude index; PRx, pressure reactivity index; RAC, correlation (R) between slow-waves of AMP (A) and CPP (C); RAP, compensatory reserve index. Bolded *p* values are those reaching statistical significance of 0.05 on Mann-Whitney *U* testing. Note: none remained significant after correction for multiple comparisons using Bonferroni methodology.

Within the 1st 72 h data set, a statistically significant difference was found for mean ICP, % time ICP >20 mm Hg, % time ICP >22 mm Hg, mean CPP, and % time CPP >70 mm Hg, where females showed higher ICP (*p* = 0.007), % time ICP >20 mm Hg (*p* = 0.012), % time ICP >22 mm Hg (*p* = 0.010), lower mean CPP (*p* = 0.016) and % time with CPP >70 mm Hg (*p* = 0.007). Of note, all relationships were statistically insignificant when adjusting for multiple comparisons. All other variables yielded insignificant statistical findings in both datasheets. This includes cerebrovascular reactivity indices, where none of PRx, PAx, or RAC demonstrated a significant difference based on biological sex.

### Age as a continuous variable—regression analysis

**FIG. 2.**
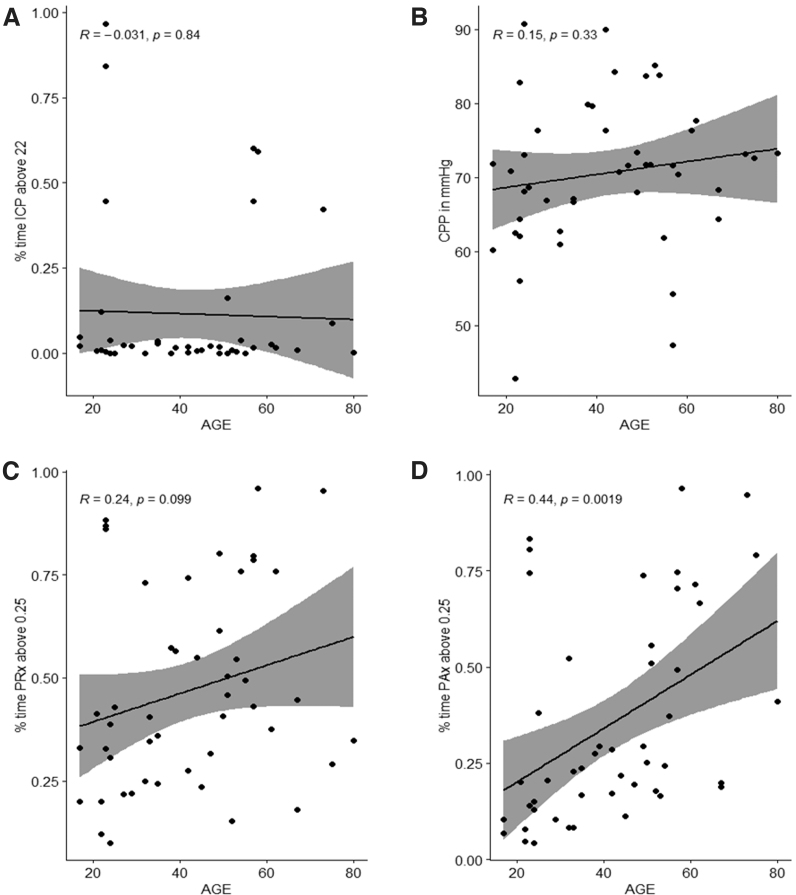
Scatterplots and linear regression (first 72 h of recording): Panel A, % time with intracranial pressure (ICP) >22 mm Hg vs. age; Panel B, mean cerebral perfusion pressure (CPP) vs. age; Panel C, % time with pressure reactivity index (PRx) > +0.25 vs. age; Panel D, % time with PAx > +0.25 vs. age. R = Pearson correlation coefficient. The *p* values recorded are for the Pearson correlation coefficient.

Next, we evaluated the relationship between cerebrovascular reactivity and age using linear regression techniques. Our findings here suggested that PRx variables demonstrated no relationship with age in our cohort while AMP-derived metrics, such as PAx and RAC, demonstrated a statistically significant linear relationship with age. As an exemplar, Figure 2 displays the scatterplots and linear regressions presented with 95% CI for the 1st 72 h of recording, with similar findings for the entire recording period displayed in Supplementary Appendix SA2. Figure 2 displays age relationship to % time ICP >22 mm Hg, mean CPP, % time PRx >0.25, and % time PAx >0.25.

Figures 2A, B, and C show no statistically significant change in the physiology variables with increasing age, with relatively unchanged ICP, CPP, or PRx across the age range using Pearson correlation. By Kendall's tau correlation, Supplementary Appendix SA2, page 2, Figure C shows a statistically positive linear relationship between % time PRx >0.25 and age (R = 0.2, *p* = 0.04). Figure 2D shows a statistically positive linear relationship between % time with PAx > +0.25 and age (Pearson correlation = 0.4; *p* = 0.002, Kendall tau correlation = 0.4; *p* = 0.0002).

### Age categorical analysis

Given the interesting results of the linear regression analysis with respect to PRx, PAx, and RAC, we decided to explore the relationship between age and cerebrovascular reactivity through the categorical organization of age. To assess the relationship between age categories and cerebral physiological response, we conducted the analysis using both a dichotomization and trichotomization of age, as detailed in the Methods section. Using dichotomized age categories (age <60 and age ≥60), there was not a reproducibly significant relationship between any of the cerebral physiological variables and age categories evident in both data sheets.

Table 4 provides the trichotomization analysis for the 1st 72 h of recording, highlighting medians/IQRs across each age group and the Kruskal-Wallis *p* values. Data for the trichotomization analysis for the entire recording period can be found in Supplementary Appendix SA3, with similar findings for both the 1st 72 h and entire recording periods. When age was trichotomized (i.e., age <40, age 40–60, and age >60), both data sheets demonstrated a statistically significant worsening of cerebrovascular reactivity, as assessed by PAx and RAC, with increasing age category, as assessed through Kruskal-Wallis testing. It was the case for mean PAx (*p* = 0.042), % time PAx >0 (*p* = 0.015), % time PAx > +0.25 (*p* = 0.013), mean RAC (*p* = 0.029), and % time RAC > -0.10 (*p* = 0.047), in the 1st 72 h recording sheet. When multiple comparisons were corrected for (*p* < 0.001), however, no values were statistically significant.

**Table 4.** Cerebral Physiology Association with Trichotomized Age (Age <40, Age 40–60, Age >60): Medians/Interquartile Ranges and Kruskal-Wallis Testing of the First 72 Hours of Recording

Physiological variable	Age <40 years (*n* = 24)		Age 40–60 years (*n* = 18)		Age >60 years (*n* = 7)		Kruskal-Wallis test *p*
	Median	IQR	Median	IQR	Median	IQR	
ICP (mm Hg)	11.9	8.8–13.3	9.8	7.1–12.6	7.9	6.7–12.1	0.487
% Time ICP >20 mm Hg	5.3	0.0–7.3	1.9	0.9–4.8	2.3	1.8–8.5	0.824
% Time ICP >22 mm Hg	2.1	0.0–3.7	1.5	0.4–3.8	1.6	0.9–5.6	0.915
MAP (mm Hg)	81.4	76.1–87.1	83.0	79.2–90.5	78.3	76.9– 83.5	0.411
CPP (mm Hg)	67.1	62.6–73.1	71.7	70.4–83.8	73.2	70.5–74.8	0.196
% Time CPP >70 mm Hg	42.2	33.8–64.6	65.2	52.7–74.7	71.7	54.4–77.0	0.102
% Time CPP <60 mm Hg	11.8	7.1–19.1	6.0	2.3–13.7	64.6	2.6–19.8	0.328
PRx (a.u.)	0.107	0.042–0.215	0.241	0.127–0.424	0.135	0.075–0.301	0.264
% Time PRx >0	58.2	49.4–78.7	74.3	63.9–82.6	63.8	57.0–80.6	0.283
% Time PRx >0.25	34.5	23.1–49.7	52.5	41.4–75.4	37.6	31.9–60.3	0.122
% Time PRx >0.35	25.9	16.1–36.3	39.0	32.4–65.6	23.8	20.4–52.5	0.128
PAx (a.u.)	-0.040	-0.116 -0.052	0.051	-0.040–0.265	0.370	0.058–0.458	**0.042**
% Time PAx >0	44.3	33.3–57.8	53.5	44.8–78.1	87.0	57.1–94.1	**0.015**
% Time PAx >0.25	16.8	9.4–28.5	29.0	20.0–54.5	66.7	30.5 − 75.3	**0.013**
RAC (a.u.)	-0.264	(-0.406)–(-0.103)	-0.157	-0.241–0.123	-0.017	-0.063–0.388	**0.029**
% Time RAC > -0.10	35.3	15.3–50.1	45.3	33.2–72.9	57.4	52.4–88.9	**0.047**
% Time RAC > -0.05	32.8	14.0–43.3	39.4	28.4–69.1	53.4	45.4–87.0	0.054
RAP (a.u.)	0.525	0.390 -0.676	0.646	0.517–0.745	56.4	0.342–0.736	0.428
% Time RAP >0.4	70.2	57.0 − 85.4	79.2	66.7–86.8	75.3	41.7–87.4	0.583
Best admission GCS	7.0	5.0–8.5	8.0	4.5–8.0	8.0	6.0–11.0	0.550
Best admission GCS – Motor	5.0	3.0–5.0	5.0	2.5–5.0	5.0	2.5–5.5	0.921
Rotterdam CT grade	5.0	4.0–6.0	4.0	3.3–5.0	5.0	4.0–5.0	0.652

a.u., arbitrary units; AMP, pulse amplitude of ICP; CPP, cerebral perfusion pressure; CT, computed tomography; GCS, Glasgow Coma Scale; ICP, intracranial pressure; IQR, interquartile range; MAP, mean arterial pressure; mm Hg, millimeters of mercury; PAx, pulse amplitude index; PRx, pressure reactivity index; RAC, correlation (R) between slow-waves of AMP (A) and CPP (C); RAP, compensatory reserve index. Bolded *p* values indicate those reaching statistical significance of 0.05 on Kruskal-Wallis testing. There were no statistically significant *p* values after Bonferroni correction for multiple comparisons (alpha 0.001).

## Discussion

Age and sex analysis in this cohort of patients revealed some interesting findings. Overall, physiology results shown in Table 2 were relatively similar for both data sheets showing that patients maintained fairly stable levels without major fluctuations for the duration of their ICU stay. Further, from the box plots in Figure 1, acute management involving ICP and CPP were adequate in keeping with the BTF guideline-based ranges.^25^ Finally, the data demonstrated that PRx insult burden was substantial over the course of the first few days of ICU stay and remained relatively independent to ongoing active treatment, which validates previous findings from the Collaborative European NeuroTrauma Effectiveness Research in TBI study.^12,46^ Yet, aside from these promising findings, there were some additional findings of interest that deserve highlighting.

First, analysis based on biological sex failed to demonstrate statistically significant differences between males and females with respect to impaired cerebrovascular reactivity, which was demonstrated through analysis of PRx, PAx, and RAC-based measures. Results from biological sex analysis, however, did show statistical significance where females had higher mean ICP, % time ICP >20 mm Hg, % time ICP >22 mm Hg, and lower mean CPP and % time CPP >70 mm Hg. The previous was noted despite females displaying similar admission CT injury burden scores as males.

In keeping with the literature, we know that in the face of high ICPs, we tend to see low CPPs (CPP = MAP–ICP), and it stands out as significant in this patient population.^22,46^ Thus, the high ICP and low CPP values in our cohort were seen among the females who are younger instead of the older male patients, which is similar with a single study on sex-based analysis of high-frequency cerebral physiology in moderate/severe TBI.^19^ Our results differ, however, because cerebrovascular reactivity metrics do not appear to be different between males versus females, as seen in this previous work.^19^

The findings here do deserve more detailed exploration in larger multi-center cohorts with high-frequency cerebral physiological data, because we had a small female population of seven patients in this cohort. Subsequently, no definitive conclusions regarding the relationship between biological sex and cerebrovascular reactivity can be made from our analysis. As such, the relationship of sex on cerebral physiology is still unclear in the literature^47^ and will remain a focus of ongoing large multi-center collaboratives.^48,49^

Second, our analysis of the impact of age on cerebrovascular reactivity has led to important insights into differences between ICP-derived index measures. In support of our hypothesis of a maturation-degenerative process of the cerebral vessels, we see AMP-derived cerebrovascular reactivity metrics PAx and RAC worsen with increasing age.^18,36,50^ From our regression analysis and trichotomization of age analysis, it is indeed seen that a statistically significant relationship was evident showing worsening of cerebrovascular reactivity with increasing age, while PRx and acute management indices did not reflect this.^51^ This is in contrast to previous work in the area, demonstrating advanced age leading to worse PRx.^19^ Although it must be acknowledged that ICP/CPP derangements in the historical cohort of these previous publications were much more prevalent than in our cohort,^19^ potentially driving the PRx derangements seen.^27,46,52,53^

The poor performance of the “standard” PRx metric in detecting impaired cerebrovascular reactivity in our cohort raises important questions regarding optimal cerebrovascular reactivity indices for subpopulations of patients with TBI. The PRx performed poorly in detecting deranged cerebrovascular reactivity in older persons, with none of the PRx metrics displaying statistically significant relationships with age, while PAx and RAC displayed this relationship. It can likely be attributed to brain volume loss at advanced ages, because brain atrophy does not allow for adequate swelling and generation of critical ICPs, which is a known driver of autoregulation failure in moderate/severe TBI.^27,46,52,53^

Further, it is supported by our cohort having aggressive ICP/CPP-directed therapy, as mentioned above. Therefore, other metrics like PAx might be better at detecting impaired cerebrovascular reactivity in these situations, as suggested by the limited previous literature on PAx,^51,54,55^ and now further supported for PAx and RAC based on this study's findings. This is an important and novel finding, because it suggests that continuous bedside cerebrovascular reactivity monitoring in TBI may necessitate the use of specific indices for certain subpopulations. The findings within this study do remain preliminary and exploratory, requiring validation in larger multi-center cohorts.

All in all, some quite useful preliminary information has emerged from our analysis thus far surrounding age and sex effects. With more research on this topic and index-specific subgroup utility, more useful information will become available to aid with improving neurotrauma management and facilitate the ongoing transition toward individualized precision medicine in moderate/severe TBI. Future work in this area will require larger multi-center high-frequency physiological data sets from patients with moderate/severe TBI.^49,50^ Further, a more detailed analytics strategy, evaluating sex and age subgroups, using machine learning techniques, may shed further light on nuanced subgroup differences.^56,57^

In addition, such data sets will require integrating pediatric high-frequency physiological data to confirm the overall hypothesis of cerebrovascular control displaying an age-related maturation phenomenon. Because pediatric data sets are hard to come by, this will necessitate multi-center coordination between specialized centers of excellence in pediatric head trauma care.^58–61^ Further, epigenomics, when linked with such cerebral physiology, may demonstrate the true link between biological age, as highlighted through methylation profiles, and impaired cerebrovascular reactivity in moderate/severe TBI.

Finally, compiling high-frequency cerebral physiology with other “omics” data, such as serial proteomics, metabolomics, neuroimaging, and genotyping, may enable uncovering molecular pathways leading the cerebrovascular dysfunction in subgroups based on age and biological sex.^11,42,62^ If such molecular pathways are uncovered, they are poised to produce precision therapeutic targets aimed at the prevention and treatment of impaired cerebrovascular reactivity in moderate/severe TBI.

### Limitations

Despite the interesting and novel findings of this analysis, there are some important limitations of this investigative work that we must highlight. First, age and biological sex analysis in patients with moderate to severe TBI have not been studied in depth across the literature but rather glossed over providing gross averages of data across extended periods of recordings.^13,32^ This has been confirmed by a recent review conducted by our group on the topic, because it is related to all multi-modal cerebral physiology in moderate/severe TBI (under review). Although the results of our work do provide a step forward in terms of our understanding of the impact of age on cerebrovascular reactivity, utilizing different periods of analysis and insult burden metrics for impaired cerebrovascular reactivity, they too, are quite preliminary and require much further validation.

Second, the sample size for our study is small, which indicates low statistical power and can affect discovery rates and estimations of various effects noted in the work. This is despite our study cohort size being above the bare minimum for such high-frequency cerebrovascular physiological work.^63^ This is further emphasized by a loss of statistical significance of our findings, when adjusting for multiple comparisons using Bonferroni methodology. Further, comparing the duration of recording time between the 1st 72 h and the entire recording periods, our small cohort did not display substantial differences.

As such, definitive comments on whether there is a true difference between “acute” (i.e., 1st 72 h) physiology versus the entire period are limited at this time, with findings in our study to be considered as exploratory at this time. Moving forward, a larger sample size will help in rectifying these and other issues and is the focus of future works from our group. Further, such large multi-center data sets will facilitate more detailed subgroup analysis.

Third, it is important to note the heterogeneous population demographics. As moderate/severe TBI is a well-known heterogeneous entity and the patients in this study were actively treated, the results of this study should be taken as purely investigative. Variation in patient demographics, injury patterns, and treatments received could drive changes in the recorded cerebral physiology, and thus the relationships documented between sex and age in this work.

We must acknowledge, however, that cerebrovascular reactivity monitoring has failed to demonstrate any significant responses to active treatment in moderate/severe TBI.^12–16^ Similarly, although intracranial injury burden and pattern appear to drive impaired cerebrovascular reactivity in these patients,^33,64–66^ our cohort did not display significant differences in admission CT injury scores between subgroups analyzed. Moving forward, however, to mitigate such confounders, much larger data sets with multi-variable modeling of the relationships between cerebrovascular reactivity and age/sex will be required.

Finally, another potential issue to note is the consistently smaller number of females with moderate to severe TBI across the literature.^9,18,19,36,67^ Because males are predominant in this group of patients, they tend to dominate most subanalyses of patients in studies, which can give the impression that males have more unfavorable outcomes or are numerous in various categories.^9,18,19,36,67^ Our results, which remain exploratory, indicate a potential signal toward differences in biological sex and cerebral physiological responses with respect to ICP control. Given the small numbers of females in our cohort, however, in keeping with other TBI studies, we cannot be definitive on these findings at this time. Again, as mentioned above in the future directions section, large multi-center data sets will be required to rectify such sample size issues.

## Conclusions

Increasing age is associated with worse cerebrovascular reactivity measures in moderate/severe TBI. The PRx performed poorly to detect impaired cerebrovascular reactivity in older adults with controlled ICP/CPP. Pulse amplitude of ICP-based indices, PAx and RAC, demonstrated an age-related deterioration in cerebrovascular reactivity in older adults with moderate/severe TBI. Therefore, it suggests the need for future work investigating specific cerebrovascular reactivity index subgroup utility, because a “one-index fits all” approach may be insufficient in detecting cerebral physiological insult burden. Biological sex did not demonstrate significant differences in measures of cerebrovascular reactivity. Additional work is required in this area.

## Supplementary Material

Supplemental data

Supplemental data

Supplemental data

## Supplementary Material

Supplementary Appendix SA1

Supplementary Appendix SA2

Supplementary Appendix SA3

## Abbreviations Used

a.u.: arbitrary units
ABP: arterial blood pressure
AMP: pulse amplitude of ICP
BTF: Brain Trauma Foundation
CI: confidence interval
CPP: cerebral perfusion pressure
CSV: comma-separated values
CT: computed tomography
EDH: epidural hematoma
GCS: Glasgow Coma Scale
ICM+: intensive care monitoring ″plus″ software
ICP: intracranial pressure
ICU: intensive care unit
IQR: interquartile range
MAP: mean arterial pressure
MMM: multi-modal monitoring
PA_x_: pulse amplitude index
PR_x_: pressure reactivity index
R: Pearson correlation coefficient
RAC: correlation between AMP and CPP
RAP: correlation between AMP and ICP
SD: standard deviation
SICU: surgical intensive care unit
TBI: traumatic brain injury
tSAH: traumatic subarachnoid hemorrhage
