# Supplementary material for: Impact of Age and Biological Sex on Cerebrovascular Reactivity in Adult Moderate/Severe Traumatic Brain Injury: An Exploratory Analysis
Source: Neurotrauma Rep. 2021 Nov 9;2(1):488–501. doi: 10.1089/neur.2021.0039 (PMC8655816; doi:10.1089/neur.2021.0039)
Supplement: Supplemental data [file Supp_AppS1.docx]

**<TT>Supplementary Appendix SA1. Cerebral Physiology Based on Biological Sex: Medians/Interquartile Ranges and Mann-Whitney *U* Testing of the Entire Recording Period**

| ***<TCH>Physiological variable*** | ***Males (*n *= 42)*** | | ***Females (*n *= 7)*** | | ***Mann-Whitney-U test***  **p** |
| --- | --- | --- | --- | --- | --- |
|  | ***Median*** | ***IQR*** | ***Median*** | ***IQR*** |  |
| <TB>ICP (mm Hg) | 9.5 | 7.1–13.3 | 18.5 | 12.0–22.9 | **0.010** |
| % Time ICP >20 mm Hg | 2.3 | 0.3–7.2 | 38.0 | 6.8–82.6 | **0.015** |
| % Time ICP >22 mm Hg | 1.6 | 0.2–4.2 | 29.8 | 3.1–77.4 | **0.015** |
| MAP (mm Hg) | 80.9 | 77.4–88.0 | 81.9 | 80.8–86.2 | 0.812 |
| CPP (mm Hg) | 71.2 | 68.2–77.0 | 60.7 | 56.8–68.9 | 0.053 |
| % Time CPP >70 mm Hg | 61.2 | 42.9–73.9 | 33.4 | 22.9–52.1 | **0.026** |
| % Time CPP <60 mm Hg | 9.9 | 2.9–17.6 | 21.4 | 11.6–34.2 | 0.086 |
| PRx (a.u.) | 0.150 | 0.030–0.278 | 0.073 | 0.021–0.637 | 0.886 |
| % Time PRx >0 | 68.5 | 51.3–82.8 | 57.4 | 49.2– 85.2 | 0.909 |
| % Time PRx >0.25 | 41.4 | 24.9–57.3 | 34.5 | 26.1–81.1 | 0.842 |
| % Time PRx >0.35 | 31.9 | 17.0–45.1 | 24.6 | 18.7–78.1 | 0.753 |
| PAx (a.u.) | 0.018 | -0.098–0.210 | 0.017 | -0.026–0.442 | 0.510 |
| % Time PAx >0 | 51.4 | 36.1–70.8 | 53.8 | 45.0–81.3 | 0.587 |
| % Time PAx >0.25 | 24.4 | 15.2–48.0 | 27.3 | 20.6–71.1 | 0.474 |
| RAC (a.u.) | -0.191 | (-0.313)–(-0.029) | -0.192 | -0.355–0.283 | 0.819 |
| % Time RAC > -0.10 | 40.6 | 27.0–60.4 | 38.5 | 25.6–79.1 | 0.864 |
| % Time RAC > -0.05 | 36.9 | 23.3–55.8 | 33.9 | 22.4–76.9 | 0.797 |
| RAP (a.u.) | 0.592 | 0.406–0.736 | 0.695 | 0.560–0.802 | 0.170 |
| % Time RAP 0.4 | 75.9 | 56.6–85.0 | 85.1 | 73.1–91.8 | 0.170 |
| Age (years) | 44.5 | 27.5–54.8 | 24.0 | 23.0 –34.0 | 0.092 |
| Best admission GCS | 7.0 | 5.3–9.0 | 7.0 | 3.0–7.5 | 0.267 |
| Best admission GCS – Motor | 5.0 | 3.0–5.0 | 5.0 | 1.0–5.0 | 0.572 |
| Rotterdam CT Grade | 4.5 | 4.0–5.0 | 5.0 | 4.0–5.5 | 0.638 |

<TFN>a.u., arbitrary units; AMP, pulse amplitude of ICP; CPP, cerebral perfusion pressure; CT, computed tomography; GCS, Glasgow Coma Scale; ICP, intracranial pressure; IQR, interquartile range; MAP, mean arterial pressure; mm Hg, millimeters of mercury; PAx, pulse amplitude index; PRx, pressure reactivity index; RAC, correlation (R) between slow-waves of AMP (A) and CPP (C); RAP, compensatory reserve index. Bolded *p* values are those reaching statistical significance of 0.05 on Mann-Whitney *U* testing. Note: none remained significant after correction for multiple comparisons using Bonferroni methodology.
