# Supplementary material for: Impact of Age and Biological Sex on Cerebrovascular Reactivity in Adult Moderate/Severe Traumatic Brain Injury: An Exploratory Analysis
Source: Neurotrauma Rep. 2021 Nov 9;2(1):488–501. doi: 10.1089/neur.2021.0039 (PMC8655816; doi:10.1089/neur.2021.0039)
Supplement: Supplemental data [file Supp_AppS2.docx]

Supplementary Appendix SA2.

Scatterplots with 95% confidence interval between age and % time ICP > 22 mm Hg, mean CPP, % time PRx > 0.25 and % time PAx > 0.25 of the entire recording period


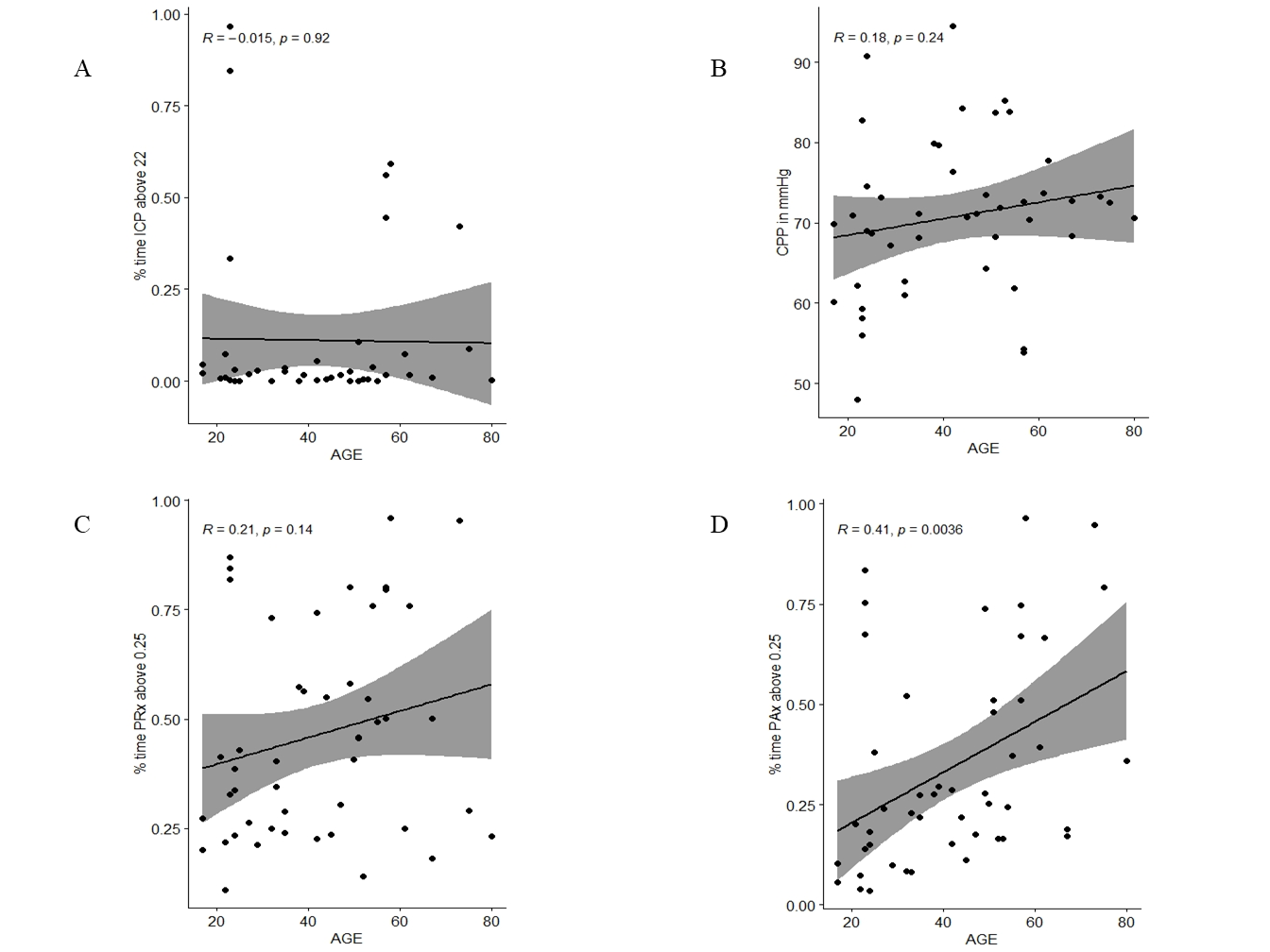


p = p-value, R = Pearson Correlation Coefficient. Panel A - % Time with ICP >22 mm Hg vs. Age, Panel B – Mean CPP vs. Age, Panel C - % Time with PRx > +0.25 vs. Age, Panel D - % Time with PAx > +0.25 vs. Age. P-values recorded are for the Pearson correlation coefficient.

Scatterplots with 95% confidence interval between age and % time ICP > 22 mmHg, mean CPP, % time PRx > 0.25 and % time PAx > 0.25 of the 1^st^ 72 hours of recording – Kendall’s Tau Correlation


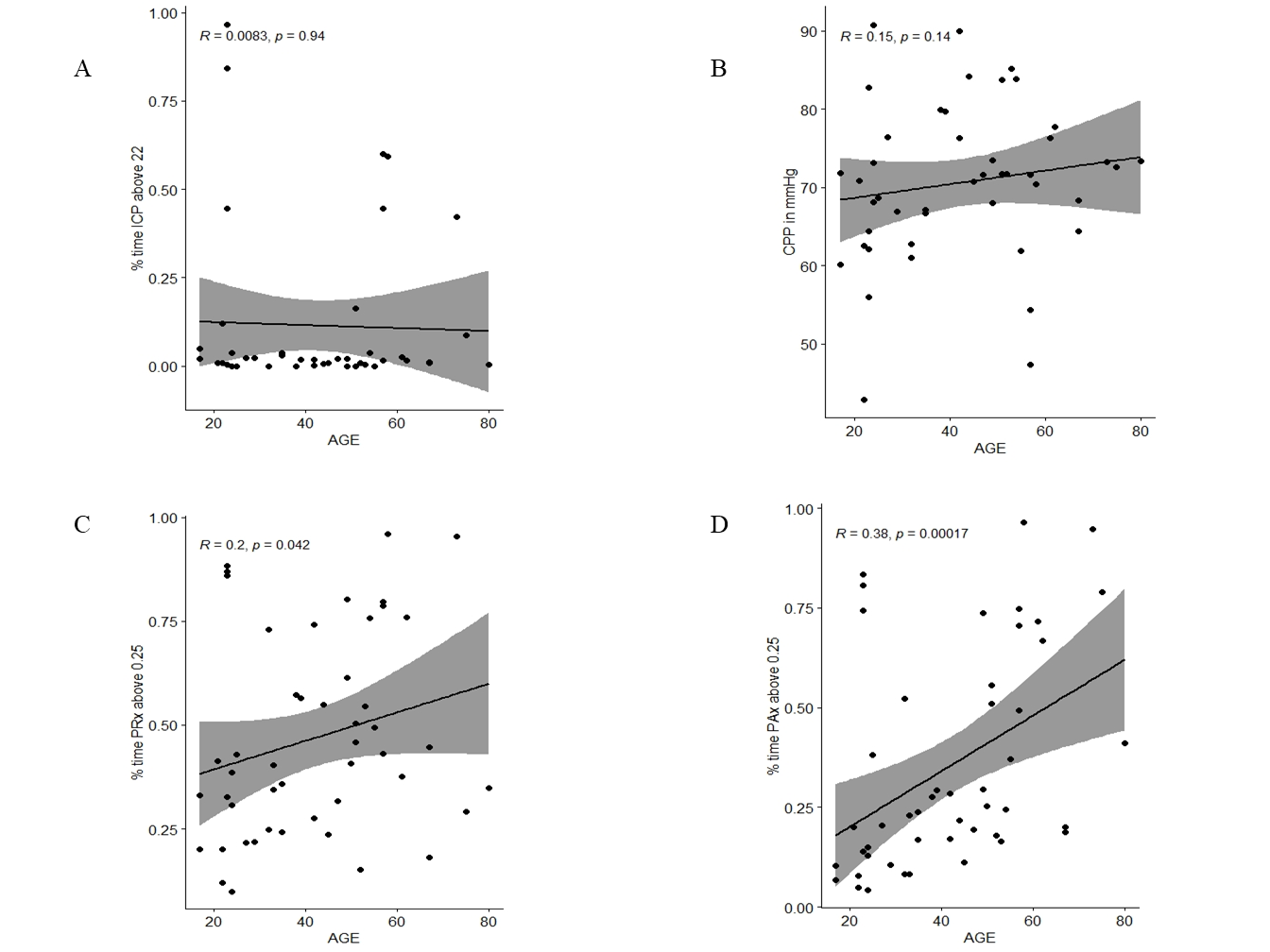


*p = p-value, R = Kendall’s Tau Correlation Coefficient. Panel A - % Time with ICP >22 mmHg vs. Age, Panel B – Mean CPP vs. Age, Panel C - % Time with PRx > +0.25 vs. Age, Panel D - % Time with PAx > +0.25 vs. Age. P-values recorded are for Kendall’s Tau correlation coefficient.*

Scatterplots with 95% confidence interval between age and % time ICP > 22 mmHg, mean CPP, % time PRx > 0.25 and % time PAx > 0.25 of the Entire Recording Period – Kendall’s Tau Correlation


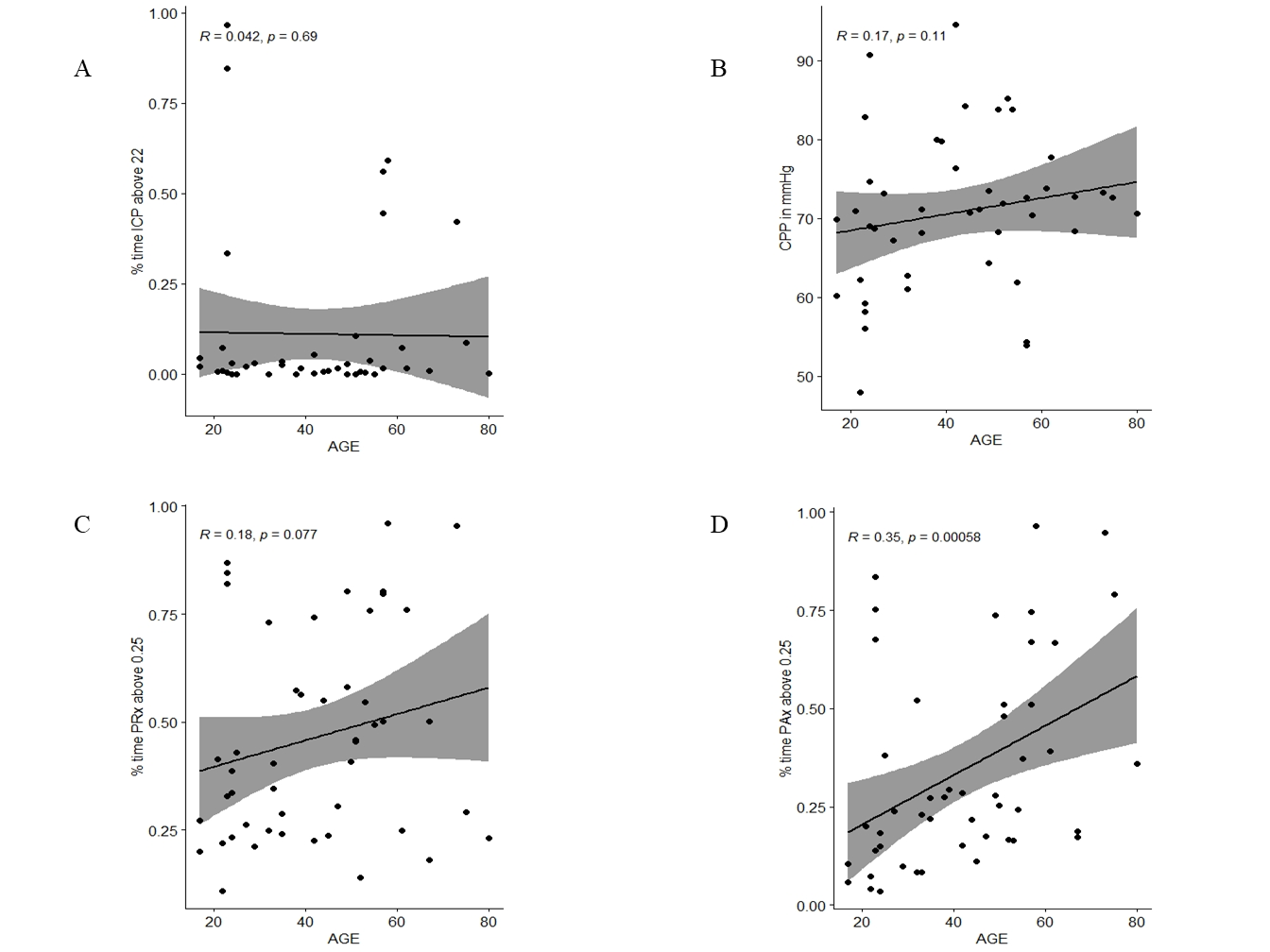
*p = p-value, R = Kendall’s Tau Correlation Coefficient. Panel A - % Time with ICP >22 mmHg vs. Age, Panel B – Mean CPP vs. Age, Panel C - % Time with PRx > +0.25 vs. Age, Panel D - % Time with PAx > +0.25 vs. Age. P-values recorded are for the Kendall’s Tau correlation coefficient.*
