# Supplementary material for: Impact of Age and Biological Sex on Cerebrovascular Reactivity in Adult Moderate/Severe Traumatic Brain Injury: An Exploratory Analysis
Source: Neurotrauma Rep. 2021 Nov 9;2(1):488–501. doi: 10.1089/neur.2021.0039 (PMC8655816; doi:10.1089/neur.2021.0039)
Supplement: Supplemental data [file Supp_AppS3.docx]

**<TT>Supplementary Appendix SA3. Cerebral Physiology Association with Trichotomized Age (Age <40, Age 40–60, Age > 60): Medians/Interquartile Ranges and Kruskal-Wallis Testing of the Entire Recording Period**

| ***<TCH>Physiological variable*** | ***Age <40 years (*n *= 24)*** | | ***Age 40–60 years (*n *= 18)*** | | ***Age >60 years (*n *= 7)*** | | **Kruskal-Wallis Test**  **p-value** |
| --- | --- | --- | --- | --- | --- | --- | --- |
|  | ***Median*** | ***IQR*** | ***Median*** | ***IQR*** | ***Median*** | ***IQR*** |  |
| <TB>CP (mm Hg) | 11.8 | 8.8–13.6 | 9.8 | 7.1–14.5 | 7.9 | 6.7–12.4 | 0.668 |
| % Time ICP >20 mm Hg | 5.1 | 0- 7.4* | 1.9 | 0.9–9.8 | 2.3 | 1.9–10.9 | 0.826 |
| % Time ICP >22 mm Hg | 2.0 | 0–3.5 | 1.7 | 0.4–5.4 | 1.6 | 1.0–8.0 | 0.808 |
| MAP (mm Hg) | 81.2 | 76.4–86.7 | 83.5 | 80.0–90.5 | 80.9 | 76.7–85.5 | 0.521 |
| CPP (mm Hg) | 68.7 | 61.0–73.2 | 71.9 | 68.2–83.8 | 72.7 | 71.6–73.5 | 0.176 |
| % Time CPP >70 mm Hg | 42.9 | 39.4–67.7 | 65.2 | 50.7–74.7 | 71.7 | 54.4–72.8 | 0.095 |
| % Time CPP <60 mm Hg | 12.4 | 7.2–21.5 | 5.5 | 2.6–18.4 | 11.6 | 6.8–15.3 | 0.335 |
| PRx (a.u.) | 0.098 | 0.032–0.215 | 0.240 | 0.145–0.424 | 0.030 | 0.004–0.328 | 0.258 |
| % Time PRx >0 | 59.7 | 49.4–78.7 | 73.7 | 65.3–83.3 | 51.8 | 49.7–78.5 | 0.217 |
| % Time PRx >0.25 | 33.6 | 24.4–49.7 | 52.3 | 42.0–75.4 | 29.1 | 24.0–63.0 | 0.183 |
| % Time PRx >0.35 | 24.4 | 17.3–36.3 | 39.4 | 32.4–65.6 | 22.7 | 14.7–55.0 | 0.202 |
| PAx (a.u.) | -0.013 | -0.135–0.059 | 0.049 | -0.045–0.230 | 0.111 | 0.032–0.456 | 0.090 |
| % Time PAx >0 | 45.2 | 33.3–58.3 | 53.5 | 44.8–77.0 | 62.0 | 52.5–90.0 | 0.059 |
| % Time PAx > 0.25 | 20.1 | 9.1–28.5 | 28.2 | 18.6–51.0 | 39.2 | 27.4–72.9 | **0.036** |
| RAC (a.u.) | -0.263 | (-0.416)–(-0.103) | -0.175 | (-0.304)–(-0.016) | -0.023 | -0.189–0.388 | 0.099 |
| % Time RAC > -0.10 | 36.5 | 16.6–50.1 | 42.9 | 28.0–64.4 | 60.4 | 38.9–88.9 | 0.183 |
| % Time RAC > -0.05 | 33.2 | 15.0–43.3 | 37.8 | 24.0–60.4 | 55.9 | 33.1–87.0 | 0.177 |
| RAP (a.u.) | 0.497 | 0.400–0.703 | 0.673 | 0.548–0.752 | 0.638 | 0.319–0.736 | 0.343 |
| % Time RAP >0.4 | 67.5 | 55.8–85.2 | 80.4 | 70.0–88.8 | 79.9 | 41.7–87.4 | 0.426 |
| Best admission GCS | 7.0 | 5.0–8.5 | 8.0 | 4.5–8.0 | 8.0 | 6.0–11.0 | 0.550 |
| Best admission GCS – Motor | 5.0 | 3.0–5.0 | 5.0 | 2.5–5.0 | 5.0 | 2.5–5.5 | 0.921 |
| Rotterdam CT grade | 5.0 | 4.0–6.0 | 4.0 | 3.3–5.0 | 5.0 | 4.0–5.0 | 0.652 |

<TFN>a.u., arbitrary units; AMP, pulse amplitude ICP; CPP, cerebral perfusion pressure; CT, computed tomography; GCS, Glasgow Coma Scale; ICP, intracranial pressure; IQR, interquartile range; MAP, mean arterial pressure; mm Hg, millimeters of mercury; PAx, pulse amplitude index; PRx. pressure reactivity index; RAC, correlation (R) between slow-waves of AMP (A) and CPP (C); RAP, compensatory reserve index. Bolded *p* values indicate those reaching statistical significance of 0.05 on Kruskal-Wallis testing. There were no statistically significant *p* values after Bonferroni correction for multiple comparisons (alpha 0.001).

****Author: Is this a hyphen (to be put in parens) or a small dash to indicate a span? Please clarify.***
